# A Review of Mixed Ionic–Electronic Conductors Oxygen Transport Membranes for Oxygen Separation: Materials, Design and Applications

**DOI:** 10.3390/ma19122477

**Published:** 2026-06-09

**Authors:** Jingjun Li, Qiangchao Sun, Hongwei Cheng

**Affiliations:** 1 School of Materials Science and Engineering, Shanghai University, Shanghai 200444, China; lijingjun@shu.edu.cn (J.L.); qiangchao_sun@shu.edu.cn (Q.S.); 2 State Key Laboratory of Advanced Refractories, Shanghai University, Shanghai 200444, China

**Keywords:** carbon capture, mixed ionic–electronic conduction, oxygen-enriched combustion, oxygen transport membranes

## Abstract

Against the backdrop of the global energy transition, novel oxygen separation technologies that combine high selectivity, high permeability, and stability have become the key to overcoming industrial bottlenecks. Mixed ion–electron conductor (MIEC) ceramic oxygen transport membranes (OTMs), with their 100% oxygen selectivity, high oxygen permeability, and low energy consumption, are regarded as the most promising next-generation oxygen separation technology. Compared with traditional oxygen production approaches including cryogenic distillation and pressure swing adsorption (PSA), these solutions make up for their inherent defects. They have extensive application prospects in oxygen-enriched combustion, CCUS, high-efficiency hydrogen preparation and chemical synthesis processes. This paper systematically reviews the progress in the oxygen transport mechanisms, material systems, structural design, and fabrication processes of MIEC oxygen permeable membranes. Finally, we conducted an in-depth analysis of the key challenges OTMs face when applied to oxygen-enriched combustion including stability in high-temperature, complex flue gas environments and the optimization of oxygen permeability and offered insights into future research and industrialization directions.

## 1. Introduction

Amid the global energy transition and the urgent need to achieve carbon peaking and carbon neutrality goals, low-carbon, high-efficiency energy production and environmental management technologies have become central focuses in both scientific research and industrial sectors [1,2]. Carbon Capture, Utilization, and Storage (CCUS) technology, as a key pathway for controlling carbon emissions from fossil fuels, relies heavily on high-purity oxygen for critical processes such as oxygen-enriched combustion, efficient hydrogen production, and chemical synthesis [3,4,5]. However, traditional oxygen production technologies face significant limitations: While cryogenic distillation yields high-purity oxygen, it is energy-intensive, equipment-complex, and costly; Pressure Swing Adsorption (PSA) and polymer membrane separation methods are constrained by oxygen purity or scalability, struggling to meet the stringent demands of industrial applications [6,7]. Consequently, developing novel oxygen separation technologies that combine high selectivity, high permeability, and stability has become crucial for overcoming industry bottlenecks [8].

Furthermore, compared to oxygen produced as a byproduct of water electrolysis, ceramic-based membranes offer several irreplaceable core advantages in the field of oxygen separation: they achieve nearly 100% oxygen selectivity through oxygen ion conduction, enabling the direct production of ultra-pure oxygen in a single step without the need for complex purification processes such as hydrogen removal, alkali removal, or drying. This eliminates the risk of explosions caused by hydrogen–oxygen mixtures and corrosion issues associated with alkaline solutions at the source [9]. Additionally, the system consumes less energy and can be coupled with waste heat recovery. With no moving parts, it features low maintenance costs and offers modular design, on-demand oxygen production, and flexibility in high-temperature applications. Capable of producing oxygen independently without relying on hydrogen production coupling, it demonstrates significant advantages in high-purity oxygen production, operational safety, cost-effectiveness, and adaptability to various operating conditions [10].

Dense inorganic ceramic oxygen transport membranes (OTMs) stand out as a highly promising next-generation technology for oxygen separation, owing to their full oxygen selectivity, excellent oxygen permeability, and low energy demand [11]. Numerous studies have focused on the optimization and development of compact membrane-based oxygen generation devices. Among them, Wang et al. [12] developed a compact oxygen generator based on self-heated mixed ionic–electronic conducting hollow fiber membranes (MIEC HFM). This device can operate at a maximum temperature of 970 °C without the assistance of an external furnace. Experimental results demonstrate that both single-fiber and multi-fiber modules achieve an oxygen permeation flux of approximately 0.7 mL·cm^−2^·min^−1^, maintaining excellent operational stability with negligible performance degradation during 150 h of long-term continuous operation. In addition, Khajryan et al. [13] proposed a novel compact oxygen separation device that adopts steam as the purge gas for oxygen extraction from air. Specifically, when the operating temperature exceeds 700 °C, the membrane enables the separation of high-purity oxygen by virtue of the oxygen partial pressure difference between the air side and the steam side. This device suffers from inevitable performance attenuation, with a 17% reduction in oxygen permeation flux after five consecutive days of operation. The oxygen transport mechanism in OTM involves complex physicochemical steps such as surface exchange and bulk diffusion. Its performance fundamentally depends on the synergistic alignment of the material’s ionic conductivity, electronic conductivity, and structural stability [14,15,16].

After decades of development, OTM materials have evolved from early electrochemical oxygen pumps and fluorite-type single-phase conductors to perovskite-type mixed conductors and dual-phase composite membrane systems. While single-phase conductors like perovskite materials exhibit high oxygen permeation flux, they generally suffer from insufficient chemical and mechanical stability, prone to phase structure destruction in complex atmospheres containing CO_2_ and CH_4_ [17]. Dual-phase membranes, through the composite design of ion-conducting and electron-conducting phases, effectively enhance stability. However, some systems still face challenges such as impaired oxygen ion transport and low permeability. Currently, achieving high oxygen permeability alongside long-term stability under complex atmospheres while simultaneously lowering operating temperatures remains the core bottleneck for the industrial application of OTMs.

**Figure 1:**
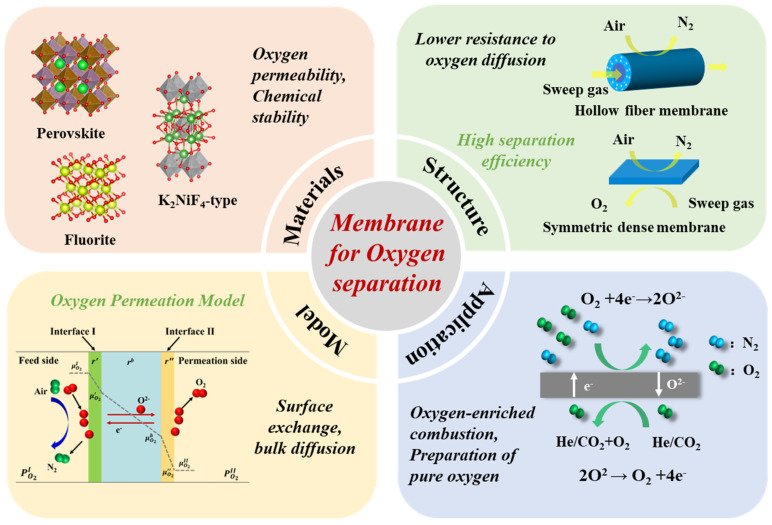
A schematic summary of the main components in this review [4].

Based on this, this paper focuses on the performance optimization and application expansion of OTM materials, systematically reviewing oxygen transport mechanisms, material system evolution, and preparation process innovations, as shown in Figure 1. By investigating the effects of ion doping, phase composition ratios, and surface modification strategies on material crystal structures, oxygen vacancy evolution, and transport kinetics, the aim is to overcome the “trade-off” effect and develop OTM materials that combine high performance with high reliability. Overall, this review is divided into three main sections. The first section explains that oxygen permeation occurs through diffusion via oxygen vacancies within the membrane; the process is jointly governed by surface exchange reactions and bulk diffusion. It introduces the concept of characteristic thickness to identify rate-limiting steps and summarizes the mainstream models of oxygen permeation flux; The second section classifies membranes by phase into single-phase membranes (fluorite-type, perovskite-type, and K_2_NiF_4_-type) and dual-phase membranes (combinations of ion-conducting and electron-conducting phases). It summarizes the doping modifications, permeation performance, and CO_2_ stability of typical materials over the past decade, noting that biphasic membranes represent the mainstream approach for balancing flux and stability; Part Three introduces three structural types: flat-sheet, tubular, and hollow-fiber. Hollow-fiber and asymmetric sandwich structures can balance permeation performance and mechanical strength. Finally, research progress is summarized, highlighting the need for future breakthroughs in the permeation-stability trade-off, high-temperature limitations, and large-scale fabrication.

## 2. Oxygen Transport Mechanism and Theory

### 2.1. Oxygen Ion Transport Process

The oxygen transport process in mixed-conductor oxygen-permeable membranes occurs through the diffusion of oxygen species via vacancies. The oxygen migration process shown above consists of the following stages:

(1) On the high oxygen partial pressure side, oxygen molecules in ambient air diffuse toward the membrane surface under oxygen concentration gradient driving force and are physically adsorbed on the material surface.
(2) These surface-active oxygen species further incorporate into the membrane lattice and exist in the form of lattice oxygen ions.
(3) Propelled by the partial pressure gradient, lattice oxygen ions move from the high-pressure side toward the low-pressure side. Meanwhile, electrons diffuse oppositely to keep the material electrically neutral.
(4) On the low oxygen partial pressure side, lattice oxygen escapes from the membrane surface through desorption and is finally released as gaseous oxygen.

When carriers are represented by oxygen vacancies and electron-hole pairs, surface exchange reactions can be expressed using Kröger–Vink notation [18], with the purging side corresponding to Equation (1) and the permeation side corresponding to Equation (2):

(1) $$\frac{1}{2}O_{2}+V_{O}^{\cdot \cdot }\to O_{O}^{\times }+2h^{\cdot }$$

(2) $$O_{O}^{\times }+2h^{\cdot }\to \frac{1}{2}O_{2}+V_{o}^{\cdot \cdot }$$

where $V_{O}^{\cdot \cdot }$, $h^{\cdot }$, and $O_{O}^{\times }$ denote oxygen vacancies, electron-hole pairs, and lattice oxygen, respectively.

### 2.2. Oxygen Transport Mechanism

Analysis of the oxygen transport mechanism reveals that the oxygen transport process involves bulk diffusion of oxygen ions and oxygen vacancies, as well as charge exchange processes at the membrane surface. The controlling factor in the overall oxygen transport process depends on the reactant with the lowest migration rate or the slowest transport process, known as the rate-determining step (RDS) [19]. During oxygen transport, bulk diffusion or oxygen surface exchange may serve as the sole RDS. However, for most oxygen transport membranes (OTMs), oxygen permeability is often jointly governed by bulk diffusion and surface exchange [20,21,22].

#### 2.2.1. Characteristic Thickness

To determine the RDS during oxygen permeation, Bouwmeester et al. [23] defined the characteristic thickness as the thickness of the oxygen-permeable membrane material at which the surface exchange reaction resistance equals the bulk diffusion resistance, as shown in Equation (3):

(3) $$L_{c}=\frac{D^{*}}{K_{S}}$$

In the equation, $D^{*}$ and $K_{S}$ represent the diffusion coefficient of oxygen ions and the oxygen surface exchange coefficient, respectively. Two key sets of factors determine the characteristic thickness: the intrinsic ratio of oxygen surface exchange to bulk diffusion, and external experimental variables such as temperature, synthesis method, oxygen partial pressure gradient and surface microstructure of the membrane [24,25,26,27]. It bears no direct relationship to oxygen permeability or oxygen ion conductivity. When the oxygen-permeable membrane thickness L > 10 *L_c_*, bulk diffusion serves as the main controlling factor for permeability. In addition, characteristic thickness, oxygen partial pressure and temperature are influential parameters that cannot be ignored. When the oxygen-permeable membrane thickness L < 10 *L_c_*, oxygen permeability is mainly governed by surface exchange reactions. At L = 10 *L_c_*, the oxygen transmission rate is jointly controlled by both factors.

#### 2.2.2. Bulk Diffusion Control

At elevated temperatures, oxygen transport membranes in hybrid conductors undergo interactions between oxygen ions and electrons due to oxygen chemical potential gradients. If the diffusion process is controlled by bulk diffusion, it can be described by the Wanger equation [25]:

(4) $$J_{O_{2}}=\frac{1}{16F^{2}L}\int_{u_{O_{2}}^{\prime \prime }}^{u_{O_{2}}^{\prime }}t_{ion}t_{e}\sigma^{tot}du_{o_{2}}$$

(5) $$t_{ion}=\frac{\sigma_{ion}}{\sigma_{ion}+\sigma_{e}}=\frac{\sigma_{ion}}{\sigma^{tot}}$$

where $J_{O_{2}}$ is oxygen permeability (mL min^−1^ cm^−2^), *F* is the Faraday constant, *L* is membrane thickness (cm), *t_e_* is electron number, $t_{ion}$ is ion conduction number, $u_{o_{2}}$ is chemical potential of oxygen (J mol^−1^), σ^tot^, σ_ion_ and σ_e_ are total conductivity, ionic conductivity, and electronic conductivity (S cm^−1^), respectively.

#### 2.2.3. Surface Exchange Control

When the oxygen ionic conductivity rises or the membrane thickness reduces to a critical level, the bulk diffusion resistance of oxygen transport membranes (OTM) can be ignored. In this case, the surface oxygen exchange reaction dominates the overall oxygen transport process and acts as the rate-limiting step. A great disparity between bulk diffusion and surface exchange kinetics leads to an obvious gradient of oxygen chemisorption, which further changes the oxygen migration mechanism. Under such circumstances, the traditional Wagner equation is no longer applicable to accurately characterize the oxygen permeation behavior. Therefore, Ishikawa et al. [28] proposed a new oxygen surface exchange mechanism:

(6) $$O_{2(g)}+S_{\left(ad\right)}⇄O_{2(ad)}$$

(7) $$O_{2(ad)}+e^{\prime }⇄{O^{\prime }}_{2(ad)}$$

(8) $$O_{2(ad)}+2e^{\prime }+2S_{\left(ad\right)}⇄2{O^{\prime }}_{\left(ad\right)}$$

(9) $${O^{\prime }}_{\left(ad\right)}+e^{\prime }⇄{O^{\prime \prime }}_{\left(ad\right)}$$

(10) $${O^{\prime \prime }}_{\left(ad\right)}+V_{O}^{\cdot \cdot }⇄O_{O}^{\times }$$

Equation (6) describes the adsorption process of oxygen molecules, while Equations (7) and (8) correspond to charge exchange, Equation (9) shows the dissociation step, and Equation (10) shows the formation of lattice oxygen.

#### 2.2.4. The Oxygen Permeation Flux Models

**Figure 2:**
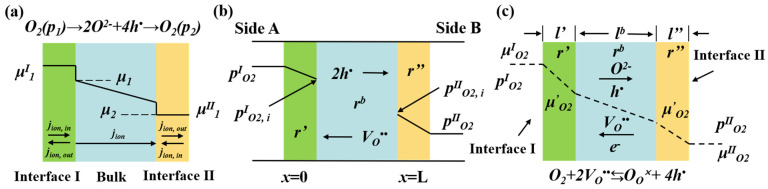
(**a**) Sketch showing oxygen permeation through ionic conductive membranes [6]; (**b**) Distribution of oxygen chemical potential across the MIEC membrane in the Kim model [6]; (**c**) Schematic representation of oxygen chemical potential decline across an MIEC membrane for Zhu’s model [6].

Oxygen permeation flux models can integrate material intrinsic parameters with actual operating conditions to quantitatively predict the oxygen permeation flux of dense oxygen-permeable membranes, effectively distinguish rate-limiting steps such as bulk diffusion and surface exchange, and elucidate the mechanisms of oxygen transport and the structure-property relationships of the materials. A common oxygen permeation model is shown in Figure 2.

Table 1 summarizes the major oxygen permeation flux models according to transport mechanisms and basic assumptions.

Macroscopic thermodynamic modeling of point defects that relates oxygen non-stoichiometry to temperature and oxygen partial pressure using the Kroger-Winkler notation and the law of mass action is a standard and experimentally validated method in this field. These point defect models are typically fitted to experimental data (such as thermogravimetric analysis or coulometric titration) to extract fundamental thermodynamic parameters, which are then used to accurately calculate and predict macroscopic oxygen permeation fluxes.

On the feed side of the membrane, oxygen molecules fill oxygen vacancies to form lattice oxygen and generate two holes; lattice oxygen diffuses into the membrane, and electrons migrate in the opposite direction to achieve charge compensation. On the permeate side, the reverse reaction occurs: lattice oxygen recombines with holes to form oxygen molecules, and oxygen vacancies are re-formed (Equation 11). The rate constants k_f_ and k_r_ characterize the forward and reverse surface exchange reactions on the two sides, respectively. The oxygen permeation flux equations incorporating these parameters are given in Equations (12) and (13) [15].

(11) $$\frac{1}{2}O_{2}+V_{O}^{\cdot \cdot }⇄O_{O}^{\times }+2h^{\cdot }$$

(12) $$J_{O_{2}}=k_{f}P_{O_{2}}^{\prime 1/2}C_{v}^{\prime }-k_{r}C_{h^{\cdot }}^{\prime 2}$$

(13) $$J_{O_{2}}=k_{f}C_{h^{\circ }}^{\prime \prime 2}-k_{f}P_{O_{2}}^{\prime \prime 1/2}C_{v}^{\prime \prime }$$

Here, C denotes the concentration of the substance indicated by the subscript, the superscript “denotes the feed side, and the subscript” denotes the permeate side of the membrane. The chemical species in Equations are denoted using Kröger–Vink notation.

## 3. Classification and Research Status

**Figure 3:**
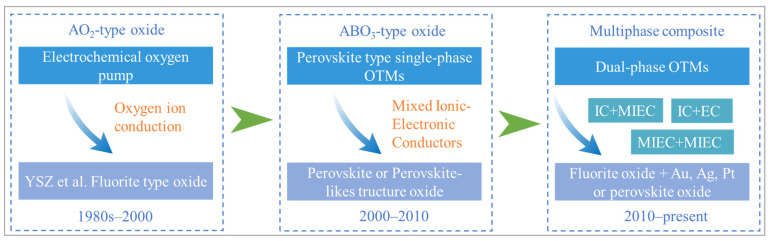
Development history of OTMs.

Oxygen transport membranes (OTMs) can be classified into two main categories based on the internal phase composition of the membrane material: single-phase mixed-conductor membranes and dual-phase mixed-conductor membranes. The development of OTM is shown in Figure 3. Among these, single-phase oxygen transport membranes can be further subdivided based on their crystal structure characteristics, primarily including fluorite-type, perovskite-type, layered perovskite-type, and K_2_NIF_4_-type structures, among others. The different phase compositions and crystal configurations directly determine the membrane material’s oxygen ion transport mechanism, permeability, and high-temperature stability. In contrast, dual-phase membranes consist of a separate oxygen-ion-conducting phase and an electron-conducting phase. These two phases complement each other’s strengths, effectively addressing the issues inherent in single-phase materials such as poor structural stability, thermal expansion mismatch, and limited conductivity, thereby enhancing the membrane material’s overall oxygen permeability and high-temperature performance. The literature review covers the past decade (2016–2026); typical single-phase OTM materials from this period are listed in Table 2, and dual-phase OTMs are listed in Table 3.

### 3.1. Single-Phase OTMs

#### 3.1.1. Fluorite-Type

Fluorite-type oxides are a class of functional ceramic materials with a typical cubic crystal structure; they contain a large number of intrinsic lattice vacancies, making it easy to create additional oxygen vacancies through ion doping. Compared to perovskite systems, fluorite-type materials exhibit greater structural stability and can operate for extended periods under high-temperature and complex atmospheric conditions. They are often modified through metal ion doping to further optimize lattice migration pathways and reduce oxygen ion diffusion resistance, thereby enhancing oxygen permeability and conductivity.

Fluorite-type oxides are generally categorized into three major families: ceria-based, zirconia-based and bismuth oxide-based materials [39]. Doping with low-valence metal ions can introduce oxygen vacancies in these systems, endowing them with prominent oxygen ionic conductivity. The development of oxygen transport membranes (OTMs) is rooted in the oxygen ion and electron conduction behaviors of electrolytes and electrodes in solid oxide fuel cells (SOFCs), where fluorite-structured oxides are widely applied as electrolytes, with yttria-stabilized zirconia (YSZ) being a typical representative.

Multiple doping strategies have been developed to optimize the performance of fluorite-structured materials. Research further reveals that gadolinia-doped ceria (GDC) possesses much higher oxygen ionic conductivity than YSZ [40]. Tuller et al. [41] first put forward the application of ceria-based oxides as solid electrolytes. It was verified that CaO-doped CeO_2_ delivers an oxygen ionic conductivity 20 times greater than CaO-stabilized ZrO_2_ at 800 °C. Sintering-aided co-doping dopants are introduced to regulate grain boundary atomic diffusion, facilitate material densification, and lower sintering temperature. CuO serves as an effective sintering additive for GDC, forming a ternary Gd_2_O_3_-CeO_2_-CuO phase to trigger liquid-phase sintering. The generated grain boundary liquid phase accelerates grain movement and rearrangement driven by capillary force, effectively improving material compactness [42]. Mixed doping of alkali metals and rare earth elements in cationic multi-element co-doping effectively cuts raw material cost while boosting ionic conductivity. Banerjee et al. [43] prepared Sm-Ca co-doped ceria, and Ce_0.8_Sm_0.05_Ca_0.15_O_1.9_ achieved a conductivity of 122 mS·cm^−1^ at 700 °C, 1.6 times higher than single Sm-doped Ce_0.8_Sm_0.2_O_1.9_. Anion-cation hybrid doping further elevates the electrical properties of fluorite materials. Wang et al. [44] fabricated F-Ca co-doped GDC series materials. The optimal Gd_0.1_Ce_0.85_Ca_0.05_F_0.1_O_1.85_ exhibited conductivity 4.2 times and 2.2 times that of pristine GDC at 600 °C and 800 °C, respectively.

#### 3.1.2. Perovskite-Type

Among various transition metal oxides, the ABO_3_-type perovskite has attracted the most research attention and seen the broadest practical applications. The A position is normally filled with large-sized divalent or trivalent rare-earth and alkaline earth metal cations, represented by La and Sr. Trivalent or tetravalent transition metal ions like Fe, Co, and Nb preferentially reside at the B position. Other metal ions with similar radii can partially replace the original cations at A and B sites without causing obvious changes to the crystal structure.

In perovskite-type materials, the B–O bond length equals a/2, while the A–O bond length is √a/2 (a denotes the lattice parameter of the cubic unit cell). The perovskite skeleton is built from eight corner-sharing BO_6_ octahedra, and A-site cations occupy the central position. Doping different elements at A and B sites will alter the structural symmetry. The tolerance factor is proposed to characterize such structural variations, and its calculation formula is presented in Equation (11):

(14) $$t=\frac{r_{A}+r_{O}}{\sqrt{2}(r_{B}+r_{O})}$$

In this formula, *r*_A_, *r*_B_, and *r*_O_ stand for the ionic radii of A-site, B-site and oxygen ions, and t denotes the tolerance factor. A value of *t* = 1 corresponds to the perfect perovskite structure. Most practical perovskites deviate from the ideal state and present structural distortion.

To achieve high performance in perovskite oxygen transport membranes (OTMs), particularly their resistance to CO_2_, doping is commonly employed. B-site doping is the most commonly studied modification strategy for improving material performance. Previous work from Shao’s group [45] revealed that a high doping content effectively improves the resistance to CO_2_ for SrFe_0.8_M_0.2_O_3−_δ films. Despite this favorable effect, the oxygen permeability of these materials declines accordingly. The doped cations studied herein include Zr^4+^, Ti^4+^, Nb^5+^, W^6+^, and Mo^6+^. Certain trivalent ion dopants with fixed valence states (such as Al^3+^, Gd^3+^, and Y^3+^) can also be used to enhance the structural stability of perovskite oxides in a carbon dioxide atmosphere. Perovskite high-valence doping improves structural and chemical stability mainly via charge compensation effect, which restrains excessive oxygen vacancy formation and suppresses reduction in B-site cations. Meanwhile, high-valent ions possess higher charge density to strengthen B-O bonding energy and lattice rigidity, moderate lattice distortion to keep stable crystal phase, raise ion migration energy barrier and hinder element segregation. It also modulates electronic structure to passivate defects, collectively enhancing thermal, redox and long-term operational stability of the material.

**Figure 4:**
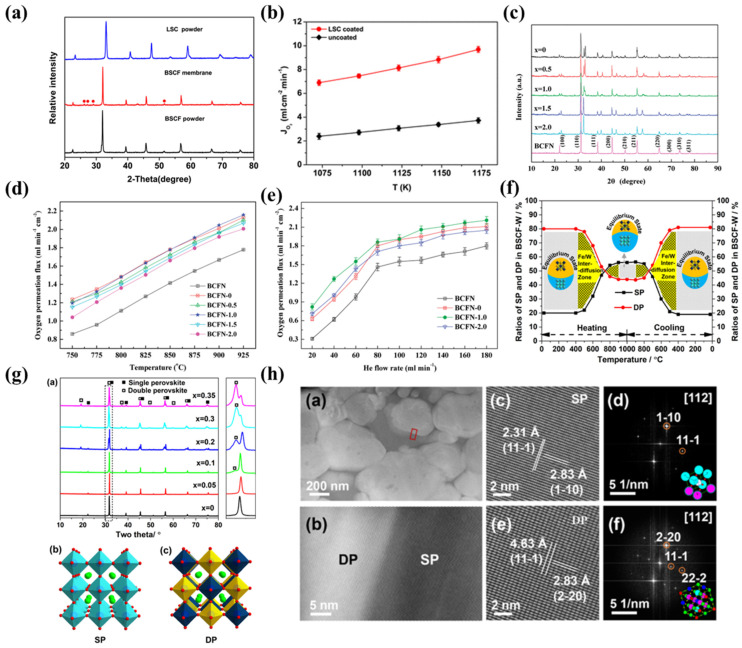
(**a**) XRD profiles of BSCF powder, BSCF membrane and LSC powder [47]. (**b**) Oxygen permeation fluxes of uncoated and LSC-coated membranes at various temperatures [47]. (**c**) XRD patterns of the BCFN membranes [48]. (**d**) Influence of coating condition and operating temperature on oxygen permeation through 1.0 mm GBCF-modified BCFN membranes [48]. (**e**) Influence of helium flow rate on oxygen permeation fluxes through 1.0 mm-thick GdBaCo_2−x_Fe_x_O_5+δ_-modified BCFN membranes [48]. (**f**) Temperature-dependent mass ratios of SP and DP phases in the BSCFW0.35 composite from Rietveld refinement [3]. (**g**) X-ray diffraction data recorded for the as-prepared Ba_0.5_Sr_0.5_(Co_0.8_Fe_0.2_)_1−x_W_x_O_3−δ_ that had been calcined at 950 °C for 10 h (The green spheres represent Ba and Sr, the green and yellow diamonds represent Co and Fe, and the black diamonds represent W) [3]. (**h**) Low-resolution HAADF-STEM micrograph of the BSFCW0.35 sample and HAADF-STEM characterization of the boundary between DP and SP crystallites in the marked zone [3].

Doping with specific ions can induce a high-temperature phase transition in perovskites, transforming them into a stable cubic phase. The optimized crystal structure effectively suppresses lattice distortion and oxygen vacancy clustering, strengthens internal chemical bonding, and enhances thermal and redox stability, enabling the material to maintain stable performance even under high-temperature and complex operating conditions. Liu et al. [46] found through high-temperature in situ XRD analysis that PSFAl_0.1_ transforms from the orthorhombic Pnma phase at 370 °C to the Imma phase, and subsequently transforms to the cubic Pm-3m phase upon heating to 720 °C. Within a certain range, Al doping improves material stability while boosting oxygen permeation performance. Perovskites not only possess oxygen transport capabilities but also exhibit certain catalytic properties. Guo et al. [47] coated the BSCF film with LSC; the XRD pattern is shown in Figure 4a. At 1173 K, the oxygen flux of the treated membrane rose obviously to 9.68 mL cm^−2^ min^−1^, as shown in Figure 4b. This is an exceptionally high value in the field of OTM. Cheng et al. [48] prepared a series of BCFN films, as shown in Figure 4c. Under helium purge, different coatings exert distinct impacts on the membrane’s oxygen permeation flux from 750 °C to 925 °C, as displayed in Figure 4d. Figure 4e further depicts the flux variation with helium flow rate at a fixed temperature of 875 °C. A notable enhancement in oxygen permeability was observed once the flow rate was elevated to 80 mL min^−1^. However, once the purge flow rate exceeded 80 mL min^−1^, the oxygen permeation flux returned to a state of slow increase. He et al. [3] reported on the development of W-doped BSCF composites, which feature two phases: Fe-rich single perovskite (SP) and W-rich double perovskite (DP), both with unique crystallographic properties. In situ high-temperature X-ray diffraction (HT-XRD) analysis was performed on BSCFW_0.35_. Using XRD data collected upon heating and cooling, the variation in the mass ratio between SP and DP phases of BSCFW0.35 with temperature was determined (Figure 4f). Figure 4g shows the room-temperature XRD pattern of the BSCFW oxide prepared after calcination at 950 °C for 10 h. For W doping levels lower than 0.1, BSCF oxide crystallizes into a single cubic perovskite structure, and no impurity diffraction peaks are observed. To clarify the structural stabilization mechanism of the BSCFW SP/DP composite at intermediate temperatures, high-resolution TEM and EDX analyses were performed on BSCFW0.35 (Figure 4h). The microstructure shows a tightly mixed two-phase structure of SP and DP, with no intermediate phase at their interface, indicating good compatibility.

#### 3.1.3. K_2_NiF_4_-Type

Numerous studies have centered on isotropic perovskite materials. In comparison, limited attention has been paid to anisotropic K_2_NiF_4_-type mixed conductors with the general A_2_BO_4_ formula. These layered composite oxides consist of alternating perovskite (ABO_3_) and rock-salt (AO) structural slabs. Site A is occupied by alkaline earth metals, rare earth elements (Ln), and other large-radius metal ions, which form a 9-coordinate complex with oxygen. Site B is occupied by a smaller-radius metal ion, typically a transition metal ion, which forms a 6-coordinate complex with oxygen, resulting in an [BO_6_] octahedral structure.

Ln_2_NiO_4+δ_ (where Ln = La, Pr, Nd, and other rare earth metals) belongs to the K_2_NiF_4_ structural family and can be used in systems such as solid oxide fuel cell cathodes and oxygen-permeable ceramic membranes. Compared to perovskite oxygen-permeable membrane materials, it offers superior stability, but has lower electronic and ionic conductivity, and requires a higher sintering temperature for the ceramic membrane. The latest research on the K_2_NiF_4_-type dense OTM series in the recent literature is (Pr_0.9_La_0.1_)_1.9_Ni_0.74_Cu_0.21_Ga_0.05_O_4+δ_. Li et al. [49] proposed a novel hierarchical porous symmetric support (GPSS) structure for K_2_NiF_4_-type (Pr_0.9_La_0.1_)_2_(Ni_0.74_Cu_0.21_Ga_0.05_)O_4+δ_ membrane, addressing the challenge of balancing high oxygen flux with long-term stability. This structure exhibits exceptional stability. At temperatures between 750 and 975 °C, the oxygen permeation rate of the PLNCG-GPSS membrane varied with feed-side oxygen partial pressure. When PO_2_ increased from 0.1 atm to 0.8 atm, the flux rose from 2.54 up to 16.47 mL min^−1^ cm^−2^. This configuration minimizes the diffusion resistance of oxide ions and boosts the surface exchange kinetics on both surfaces of the membrane.

**Table 2:** Summary of the performances of single-phase membranes.

Membrane Materials	Temp. (°C)	Flow Rate (mL min^−1^)		J_O2_ (mL min^−1^ cm^−2^)		Thickness (mm)	Ref.
		Feed	Sweep	He	CO_2_		
La_0.6_Sr_0.4_Co_0.2_Fe_0.8_O_3−δ_	900	200	100	0.81		1.25	[50]
Ce_0.8_La_0.2−x_Ti_x_O_2−δ_						0.6–0.8	[51]
SrCo_0.8−x_Fe_0.2_Mo_x_O_3−δ_	950	200	50	~1.0		1.50	[52]
Ba_0.5_Sr_0.5_(Co_0.8_Fe_0.2_)_1−x_Zn_x_O_3−δ_	700	20	120	0.65		0.40	[53]
BaBi_0.05_Co_0.8_Nb_0.15_O_3−δ_	730	200	20	15		/	[54]
(Pr_0.9_La_0.1_)_2_(Ni_0.74_Cu_0.21_Ga_0.05_)O_4+δ_Cl_0.1_	975	/	/	1.1	1.1	0.60	[55]
Sr_0.95_Ag_0.05_Co_0.9_Nb_0.1_O_3−δ_	750		100	1.14		1.00	[56]
Ba_0.5_Sr_0.5_Co_0.8_Fe_0.1_W_0.1_O_3−δ_	800			1.5		0.60	[57]
BaFe_0.95−x_Ca_0.05_Ti_x_O_3−δ_	950	120	60	1.02		0.4	[58]
SrCo_x_Fe_0.9−x_Nb_0.1_O_3−δ_	900	50	100	0.9	0.6	1.10	[59]
Pr_0.6_Sr_0.4_Cu_0.2_Fe_0.8_O_3−δ_	900		40	0.40	0.16	1.40	[24]
La_0.6_Ca_0.4_Co_1−x_Fe_x_O_3−δ_	900	150	29	0.76	0.5	1.00	[60]
Ba_0.5_Sr_0.5_Co_0.8_Fe_0.2_O_3−δ_	950	240		8		0.47	[61]
Ba_0.5_Sr_0.5_Co_0.8_Fe_0.17_Y_0.03_O_3−δ_F_0.09_	900	11	36	3.15		1.00	[62]
Ba_2_In_2−x_Cr_x_O_5−δ_	950	130	29	1.4		1.2	[63]
La_0.6_Sr_0.4_Co_0.2_Fe_0.8_O_3−δ_	850	100	100		0.876		[64]
BaCe_0.1_Fe_0.9_O_3−δ_	950	150		2.15		0.50	[65]
La_0.6_Sr_0.4_Co_0.2_Fe_0.8_O_3−δ_-Ag	950	200	50	1.48		0.30	[66]
La_0.8_Ca_0.2_Fe_0.95_Ag_0.05_O_3−δ_	950		180	1.5			[67]
(Pr_0.9_La_0.1_)_1.9_Ni_0.74_Cu_0.21_Ga_0.05_O_4+δ_	975	150	30	0.95		0.60	[68]
Pr_0.6_Sr_0.4_FeO_3−δ_	950	300	100	3.5		0.60	[69]
Ba_0.6_Sr_0.4_Fe_0.92_Ti_0.08_O_3−δ_	900	120	60	0.93		1.00	[70]
La_0.4_Bi_0.4_Sr_0.2_FeO_3−δ_	800	100	100	1.13		0.025	[71]
K_x_Ba_0.5−x_Sr_0.5_Co_0.8_Fe_0.2_O_3−δ_	750			0.80		1.00	[72]
(Pr_0.9_La_0.1_)_2_(Ni_0.74_Cu_0.21_Ga_0.05_)O_4+δ_	975	150	55	4.58		0.6	[49]
La_0.2_Pr_0.2_Nd_0.2_Ba_0.2_Sr_0.2_Co_0.8_ Fe_0.2_Ni_0.1_O_3−δ_	900	150	60		0.3	1.00	[73]
La_0.2_Pr_0.2_Nd_0.2_Ba_0.2_Sr_0.2_Co_0.8_ Fe_0.2_O_3−δ_	900	150	60	0.33	0.23	1.00	[74]
SrFe_1−x_Hf_x_O_3−δ_	850				0.36	1.00	[75]
SrCo_0.95_P_0.05_O_3−δ_Cl_0.05_	600	100	80	1.10		0.025	[48]
La_0.25_Sr_0.25_Gd_0.2_Nd_0.2_Pr_0.1_CoO_3_	950			1.62	1.46		[76]

### 3.2. Dual-Phase OTMs

Most previous studies have focused primarily on doping modifications of perovskite-based materials to improve their tolerance to carbon dioxide. In recent years, fluorite oxides with intrinsic oxygen ionic conductivity have been introduced into perovskite matrices to fabricate dual-phase membranes, offering an alternative approach to overcoming the challenges associated with perovskite membranes. This dual-purpose approach aims to enhance both oxygen permeability and stability [77].

**Figure 5:**
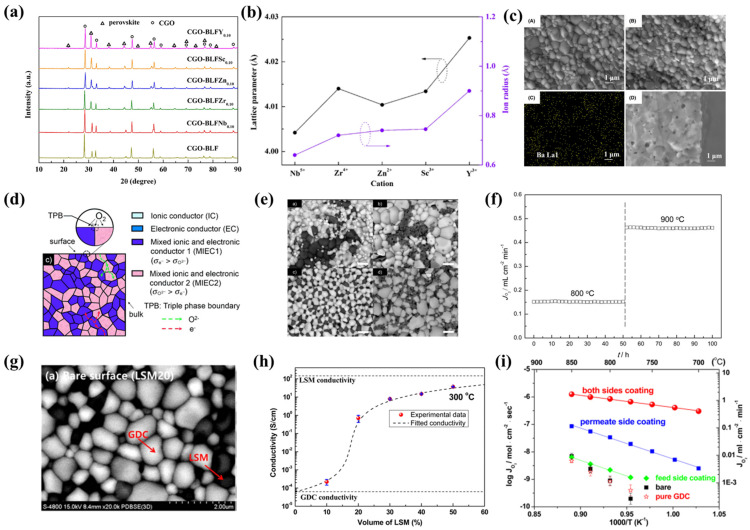
(**a**) XRD patterns of CGO-BLFM_0.10_ dual-phase; (**b**) the lattice parameters of BLFM_0.10_ [84]. (**c**) SEM-EDS images of the fresh dual-phase membranes: the surface of CGO-BLF and CGO-BLFSc0.10 [84]. (**d**) Conceptual diagram of electron and ion transport in a dual-phase membrane [81]. (**e**) Back-scattered electron (BSE) micrographs of the surfaces of biphasic membranes synthesized using different methods (One-pot method and two-pot method) and doped with copper [81]. (**f**) Stability of oxygen permeation for one-pot CGCO-LCF dual-phase membrane during long-term operation [81]. (**g**) Scanning electron micrographs of the dual-phase membrane bare surface of the LSM20 membrane [82]. (**h**) Variation in electrical conductivity of GDC/LSM dual-phase membrane with LSM content at 300 °C [82]. (**i**). Oxygen permeation fluxes measured through free-standing LSM20 dual-phase membranes with and without a porous La_0.6_Sr_0.4_CoO_3−δ_ coating [82].

Doping the fluorite phase in biphasic membranes primarily serves to enhance oxygen ion conductivity, increase oxygen vacancies, regulate thermal expansion, improve chemical stability and interfacial compatibility, and increase mechanical strength. This significantly boosts oxygen permeability while maintaining resistance to CO_2_ and reduction. Chen et al. [78] prepared a series of Ce_0.8_M_0.2_O_2−δ_-La_0.5_Sr_0.5_Fe_0.8_Cu_0.2_O_3−δ_ (M = La, Pr, Nd, Sm, Gd) biphasic membranes to investigate the effects of rare earth elements on electrical conductivity, structure, oxygen permeability, phase stability, and chemical stability in ionic conductors (cerium-doped). The high oxygen permeation flux in the CGO-LSFCO membrane can be attributed to its well-connected network, while the high electronic conductivity of the ionic conductor (CPO) and oxygen vacancy defects in the CPO-LSFCO samples may be responsible for their superior oxygen permeability. In their systematic work, Zhang et al. [79] studied how different lanthanum (Ln) elements affect the structural features and oxygen permeation performance of Ti-doped dual-phase OTMs (60wt%Ce_0.9_Ln_0.1_O_2−δ_-40wt% Ln_0.6_Sr_0.4_Fe_0.9_Ti_0.1_O_3−δ_ (CLnO-LnSFTO, Ln = La, Pr, Nd, Sm, Gd, Tb)). It benefits from the combination of fluorite phases and doping with lanthanide elements. All CLnO-LnSFTO OTMs operated stably for 50 h and 100 h, respectively, at 1000 °C under He and CO_2_ gas purging conditions, with virtually no decline in performance. In dual-phase membrane, the perovskite phase is the primary electron-transporting phase; enhancing the electronic conductivity of the perovskite helps improve the overall oxygen permeability of the dual-phase membrane. Cheng et al. [80] prepared a series of biphasic films doped at the B site of perovskites, with the composition (CGO-BLFM_0.10_, M = Fe, Nb, Zr, Zn, Sc, Y), as shown in Figure 5a. Figure 5b shows the lattice parameters of the BLFM_0.10_ oxide, which were obtained by fitting XRD data. It can be observed that, in general, the lattice parameters of BLFM0.10 increase as the radius of the dopant ion increases. The surface microstructures of CGO-BLF and CGO-BLFSc are displayed in Figure 5c. Distinct grain boundaries can be observed, with no cracks or pores existing between grains, which demonstrates the excellent densification of the prepared membranes. In a pure CO_2_ atmosphere, CGO-BLFSc0.10 exhibited the best oxygen permeation stability.

The above analysis indicates that fluorite-structured materials feature superior ionic conductivity yet inferior electronic conductivity. Accordingly, it is essential to boost the electronic conductivity of fluorite to obtain qualified MIEC oxides. Existing literature has proven that co-doping modification can effectively optimize both the electronic conductivity and sintering characteristics of fluorite oxides. When low-valent ions such as Cu^2+^ are doped into the fluorite phase, they can generate a large number of oxygen vacancies through charge conservation mechanisms, thereby increasing oxygen permeability. Zhang et al. [81] reported a new series of biphasic mixed ion–electron conductors (OTMs) composed of Ce_0.85_Nd_0.1_Cu_0.05_O_2−δ−_Nd_x_Sr_1−x_Fe_1−y_Cu_y_O_3−δ_, which exhibit oxygen permeation fluxes of 2.62 and 1.52 mL min^−1^ cm^−2^ under He and CO_2_ purging conditions, respectively, surpassing all previously reported dense dual-phase OTMs. Compared to the Ce_0.9_Pr_0.1_O_2−δ_-Pr_0.6_Sr_0.4_Fe_1−x_Ti_x_O_3−δ_ membrane (oxygen permeability of 0.512 under helium purging) previously reported by this research group, representing a fivefold increase in oxygen permeability at the same temperature and thickness. Fang et al. [82] found that doping copper into the Ce_0.9_Gd_0.1_O_2−δ_ oxide enhances both ionic and electronic conductivity, leading to a transition from ionic to mixed conductivity at high temperatures. Figure 5d illustrates the transport pathways for oxygen ions and electrons within the membrane. Figure 5e shows BSE micrographs of the surfaces of dual-phase membranes prepared using different methods, revealing that the membrane prepared using the one-pot method exhibits a uniform grain distribution. Using a 0.5 mm-thick CGCO-LCF one-pot synthesis dual-phase membrane and pure CO_2_ as the purge gas at 950 °C, an oxygen permeation flux as high as 0.70 mL · min^−1^ · cm^−2^ was achieved. The long-term oxygen permeation stability of the one-pot-derived CGCO-LCF membrane was evaluated at 800 °C and 900 °C (Figure 5f). The oxygen flux remained constant during continuous operation, confirming the favorable CO_2_ tolerance of the membrane. Joo et al. [83] investigated the optimal ratio of the two phases in GDC/LSM membranes. A scanning electron micrograph of the dual-phase membrane is shown in Figure 5g. To determine the minimum LSM content required for electroosmosis, the electrical conductivity of the GDC/LSM composite was measured as a function of its LSM content, as shown in Figure 5h. Therefore, 20 vol.% LSM was determined to be the minimum concentration required to ensure electroosmotic flow in this composite membrane. Figure 5i shows the oxygen permeation flux through a self-supporting LSM20 bimetallic membrane approximately 60 μm thick (with and without a porous LSC coating) under an air/helium gradient.

The mechanical properties of the membrane are critical for its control and amplification in commercial applications. Liu et al. [1] adopted a Vickers indenter under 0.5 kg load to characterize the mechanical properties of the as-prepared membranes. The addition of CP reduced the overall fracture toughness. CP-PSFA suffered more severe crack propagation and possessed lower fracture toughness than pristine PSFA. In addition, the smaller indentations on CP-PSFA surfaces confirmed its higher Vickers hardness.

**Table 3:** Summary of the performances of dual-phase membranes.

Membrane Materials	Temp. (°C)	Flow Rate (mL min^−1^)		J_O2_ (mL min^−1^ cm^−2^)		Thickness (mm)	Ref.
		Feed	Sweep	He	CO_2_		
Ce_0.9_Gd_0.1_O_1.95_-(La_0.8_Sr_0.2_)_0.95_MnO_3−δ_	950	100	30	0.78	0.659	0.03	[84]
Ce_0.8_Gd_0.2_O_2−δ_-Ba_0.95_La_0.05_Fe_0.9_Sc_0.1_O_3−δ_	925	300	100	0.20	0.14	1.00	[80]
Ce_0.9_Gd_0.1_O_2−δ_-La_0.6_Sr_0.4_Co_0.2_Fe_0.8_O_3−δ_	850	500	500	3.5		0.33	[85]
Pr_0.1_Gd_0.1_Ce_0.8_O_2−δ_-CoFe_2_O_4_	900	250	50	0.19		1.00	[86]
Nd_0.6_Sr_0.4_CoO_3−δ_-Ce_0.9_Nd_0.1_O_2−δ_	1000	150	60	0.9	0.55	0.40	[87]
Pr_0.6_Sr_0.4_Co_0.5_Fe_0.5−x_Nb_x_O_3−δ_-Ce_0.8_Gd_0.2_O_2−δ_	900	300	100	0.73	0.60	0.50	[20]
La_2_NiO_4+δ_-Sm_0.2_Ce_0.8_O_1.9_	950	120	120	3.05	2.25		[88]
Ce_0.9_Pr_0.1_O_2−δ_-Pr_0.6_Sr_0.4_Fe_1−x_Al_x_O_3−δ_	1000	100	49	1.12		0.40	[89]
La_0.15_Sr_0.85_FeO_3−δ_-La_0.15_Ce_0.8_Cu_0.05_O_2−δ_	950	120	60	0.65		0.6	[90]
Ce_0.8_Sm_0.2_O_2−δ_-Sr_2_Fe_1.5_Mo_0.5_O_5+δ_	900	30	30	0.20	0.16		[91]
Ce_0.9_Pr_0.1_O_2−δ_-Pr_0.6_Sr_0.4_Fe_0.8_Al_0.2_O_3−δ_	1000	150	49	1.03	0.46	0.33	[92]
Ce_0.9_Pr_0.1_O_2−δ_-Pr_0.6_Ca_0.4_FeO_3−δ_	1000	150	49	1.00	0.62	0.30	[93]
CSB_x_-Sm_0.6_Sr_0.4_Al_0.3_Fe_0.7_O_3−δ_	900	100	30	0.83		0.50	[94]
Ce_0.9_Pr_0.1_O_2−δ_-La_0.5_Sr_0.5_Fe_0.9_Cu_0.1_O_3−δ_	900	150	29	0.93	0.71	0.50	[95]
Ce_0.8_Sm_0.2_O_1.9_-La_0.8_Sr_0.2_Cr_0.5_Fe_0.5_O_3−δ_	800	200		0.97		0.05	[96]
La_0.8_Sr_0.2_MnO_3_-YSZ							[97]
Ce_0.9_La_0.1_O_2−δ_-La_0.6_Sr_0.4_Co_1−x_Al_x_O_3−δ_	1000	150	49	1.02	0.72	0.60	[98]
Ce_0.8_Sm_0.2_O_2−δ_-SrCo_0.9_Nb_0.1_O_3−δ_	1000	120	60	1.20	0.15		[99]
Ce_0.85_Pr_0.1_Cu_0.05_O_2−δ_-Pr_0.4_Sr_0.6_Fe_0.95_Cu_0.05_O_3−δ_	1000	150	49	1.60	0.98	0.60	[100]
Ce_0.8_Sm_0.2_O_2−δ_-La_0.8_Ca_0.2_Al_0.3_Fe_0.7_O_3−δ_	950	100	100	1.84	1.52	0.30	[101]
Ce_0.9_Pr_0.1_O_2−δ_-Pr_0.6_Sr_0.4_Fe_1−x_In_x_O_3−δ_	1000	150	49	1.07	0.80	0.60	[102]
Ce_0.9_Pr_0.1_O_2−δ_-Pr_0.6_Sr_0.4_Fe_1−x_Ga_x_O_3−δ_	1000	150	49	0.75	0.41	0.60	[103]
Ce_0.9_Pr_0.1_O_2−δ_-Pr_0.6_Sr_0.4_Fe_1−x_Ti_x_O_3−δ_	1000	150	49	0.512	0.306	0.60	[104]
Ce_0.8_Sm_0.2_O_2−δ_-Sm_0.6_Sr_0.4_Fe_1−x_Cu_x_O_3−δ_	900	100	40	0.48	0.32	1.00	[105]
Ce_0.9_Ln_0.1_O_2−δ_-Ln_0.6_Sr_0.4_Fe_0.9_Ti_0.1_O_3−δ_ (Ln = La, Pr, Nd, Sm, Gd, Tb)	1000	150	49	0.60	0.54	0.60	[79]
Sm_0.2_Ce_0.8_O_1.9_-La_0.9_Ca_0.1_Fe_0.95−x_Cu_x_O_3−δ_	950	100	100	1.99		0.24	[106]

In addition, the application of OTMs in membrane reactors, where the permeated oxygen is directly utilized in chemical reactions, is an emerging field of research. In particular, dual-phase OTMs in which the membrane is composed of a stable ionic conductor and a stable electronic conductor have attracted significant attention because they can overcome the drawbacks of monophasic membranes, such as mechanical stability under high-temperature and harsh operating conditions. Although considerable progress has been made in developing dual-phase OTMs for both conventional and emerging applications, multiple bottlenecks still limit their practical large-scale deployment.

## 4. Density Functional Theory Calculations

First-principles calculations are a key theoretical tool for studying the mechanisms and optimizing the performance of OTM materials. The oxygen permeability of oxygen-permeable membranes is primarily determined by the evolution of oxygen vacancies, the migration of oxygen ions between phases, and surface oxygen exchange reactions; conventional experiments struggle to directly observe these microscopic processes at the atomic scale.

**Figure 6:**
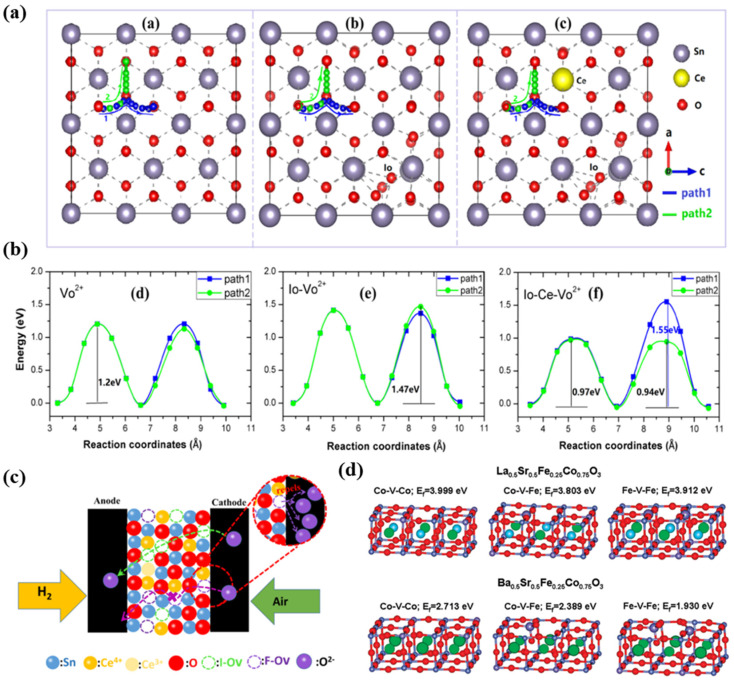
(**a**) Oxygen ion transport mechanisms of pure SnO_2_, SnO_2_ with interstitial oxygen and Ce-doped SnO_2_ [5]. (**b**) Energy transfer barriers in SnO_2_ with and without Io, and energy transfer barriers for Ce-doped SnO_2_ [5]. (**c**) Ion distribution diagram of the cell in the working state [14]. (**d**) Relaxed atomic configurations for oxygen vacancies for La_0.5_Sr_0.5_Fe_0.25_Co_0.75_O_3_ and Ba_0.5_Sr_0.5_Fe_0.25_Co_0.75_O_3_ (The green balls represent the A sites in the perovskite, the blue balls represent the B sites, and the red balls represent oxygen atoms) [14].

To elucidate the ionic conduction mechanism, in a previous study, Cai et al. [5] doped cerium into tin dioxide to prepare pure Sn_1−x_Ce_x_O_2−δ_ oxides. After combining with Sm_0.2_Ce_0.8_O_3−δ_ (SDC), the hybrid system became a semiconductor-ionic material (SIM) and was utilized as the electrolyte membrane in fuel cell assemblies. To elucidate the doping effect of Ce on oxygen ionic conduction, we adopted DFT methods to investigate pristine and Ce-substituted SnO_2_ systems. As illustrated in Figure 6a, extra ions were incorporated to remove the disturbance caused by interstitial oxygen species. The rotational and hopping energies for oxygen migration in SnO_2_ and Sn_1−x_Ce_x_O_2−δ_ were computed, and the outcomes are given in Figure 6b. It can be observed that the two oxygen migration paths in pure SnO_2_ have comparable energy barriers of roughly 1.47 eV, which are all higher than 0.94 eV. The reduction in migration energy barrier verifies that Ce doping effectively boosts oxygen ion mobility. This is the fundamental reason for the improved electrochemical behavior of fuel cells equipped with Ce-doped SnO_2_ electrolyte membranes. Figure 6c depicts the oxygen ion transport process within a fuel cell. Petkovic et al. [14] calculated the formation energy of oxygen vacancies created by removing oxygen atoms from different “O-bridges” on the perovskite surface. For most of the perovskites considered, the atoms surrounding the vacancies exhibit little relaxation and do not significantly alter the atomic configuration of the perovskite surface (Figure 6d). Across all samples, oxygen vacancy formation energies differ little between the bulk and surface regions, with surface sites exhibiting slightly lower energy.

To gain a deeper understanding of the phenomenon in which moderate Al doping simultaneously enhances both oxygen permeability and stability, Liu et al. [46] employed first-principles calculations to elucidate the formation of oxygen vacancies and the behavior of oxygen migration. Of the five oxygen migration pathways, the O1 route surrounding Al dopants in the Pr_5_Sr_3_Fe_7_Al_1_O_12_ supercell yields the minimum migration barrier of 0.67 eV, outperforming the two migration pathways in pure Pr_5_Sr_3_Fe_8_O_12_. The stronger Al–O bonding compared with Fe–O bonding stretches the neighboring Fe–O bonds. The increased Fe–O bond length enables easier oxygen ion dissociation. Qiao et al. [2] investigated the effects of co-doping with Y^3+^ and W^6+^ on the electronic structure. The PDOS values do not cross the Fermi level, indicating that both structures exhibit semiconductor behavior. Due to W doping, Ba_8_Co_6_W_1_Y_1_O_24_ has a narrower bandgap than Ba_8_Co_6_Y_2_O_24_. A narrower bandgap implies stronger electronic conductivity, suggesting that co-doping enhances electronic conductivity. Wei et al. [75] investigated the effect of Hf^4+^ doping on the mobility of oxygen ions. The calculated electron density maps of SF and Hf-doped SFHf samples on the (100) plane. Clearly, compared to the SF sample, the SFHf sample exhibits an increased electron density around the O ion center, while the density of Fe cations is reduced. Therefore, the incorporation of Hf^4+^ cations into the SF sample renders the O sites more active. When CO_2_ is used as the sweep gas, its adsorption behavior on the membrane surface is critical.

By employing first-principles methods such as density functional theory, it is possible to accurately calculate the formation energy of oxygen vacancies and the migration energy barriers of oxygen ions, identify the optimal pathways for ion transport and the rate-limiting steps of reactions, and deeply elucidate the intrinsic mechanisms of oxygen transport in these materials. The oxygen migration energy derived from theoretical calculations reflects the difficulty of oxygen ion diffusion within the crystal lattice. A lower migration energy barrier facilitates oxygen transport, resulting in higher oxygen permeability observed in experiments. In this way, the computational results provide a reasonable explanation for and support the experimental oxygen permeability.

## 5. Design of Membrane Structures

**Figure 7:**
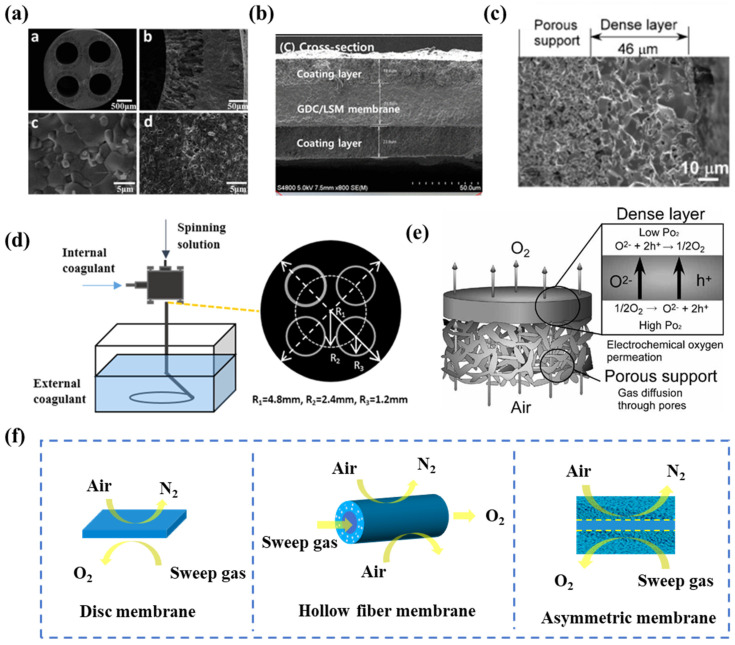
(**a**) SEM micrographs of uncoated and LSC-modified BSCF hollow fiber membranes [47]. (**b**) Cross-sectional SEM micrographs of the LSM20 membrane with LSC layers. (**c**) Cross-sectional morphology of the fabricated asymmetric BLF membrane [82]. (**d**) Schematic illustration of the phase inversion process [47]. (**e**) Schematic drawing of a dense/porous asymmetric membrane [47]. (**f**) Structural comparison diagrams of graded porous supported symmetric, hollow fiber, and asymmetric dense membranes.

Three primary structural morphologies are commonly adopted for dense inorganic oxygen transport membranes: planar, tubular, and hollow fiber geometries. Although flat-sheet membranes are relatively easy to manufacture, their small surface area limits their sealing performance. The tubular La_0.6_Sr_0.4_Co_0.2_Fe_0.8_O_3−δ_ membranes prepared by Li et al. [107] resolved the sealing issue; however, due to their relatively large thickness, they have a small specific surface area and poor oxygen permeability. Compared to sheet membranes and tubular membranes, hollow fiber membranes overcome the challenge of sealing while offering a large surface area and thin walls; as a result, under identical test conditions, their oxygen permeability is significantly higher than that of the other two membrane types. Guo et al. [47] modified the surface of BSCF mixed-conductor multi-channel hollow fiber (MCMHF) membranes by spray-coating them with a porous layer of LSC, thereby improving their oxygen permeability, as shown in Figure 7a,d.

In addition to fabricating membranes with a hollow-fiber structure, creating disc-shaped membranes with an extremely thin, asymmetric structure can significantly increase oxygen permeability without compromising the mechanical strength of the membrane. Although disc-sheet membranes have a lower specific surface area than hollow-fiber membranes, when configured in a “sandwich” structure, they offer higher mechanical strength and superior stability and durability compared to hollow-fiber membranes. The asymmetric membrane prepared by Joo et al. [82] is shown in Figure 7b. A micrograph of the 30-μm-thick self-supporting LSM20 membrane coated with LSC clearly shows that the LSC layer is porous and approximately 20 μm thick. The asymmetric films prepared by Watanabe et al. [101] are shown in Figure 7c,e, with the dense layer having a thickness of approximately 46 μm. For the porous support layer, numerous pores measuring approximately 1–2 μm were observed, indicating that the porous morphology did not degrade even after the dense layer was deposited at high temperatures.

Nam et al. [108] achieved a 24-fold increase in oxygen permeability flux in the LSGM-LSCF composite membrane compared to the uncoated membrane by applying an LSCF active coating to both sides of the composite membrane. A unique hierarchical GPSS structure was proposed by Chen et al. [109] for K_2_NiF_4_-based PNM05 membranes. Composed of a thin dense film and a porous supporting layer, the GPSS structure improves gas permeation and structural robustness. The oxygen permeability Arrhenius curve of PNM05-GPSS demonstrates a low apparent activation energy. Such a low E_a_ is ascribed to the distinctive GPSS configuration, which optimizes both bulk oxygen diffusion and surface reaction kinetics. This structure effectively shortens the diffusion path and reduces polarization effects, thereby enhancing efficiency at high temperatures. Figure 7f shows the three most common types of membrane structures currently in use.

**Figure 8:**
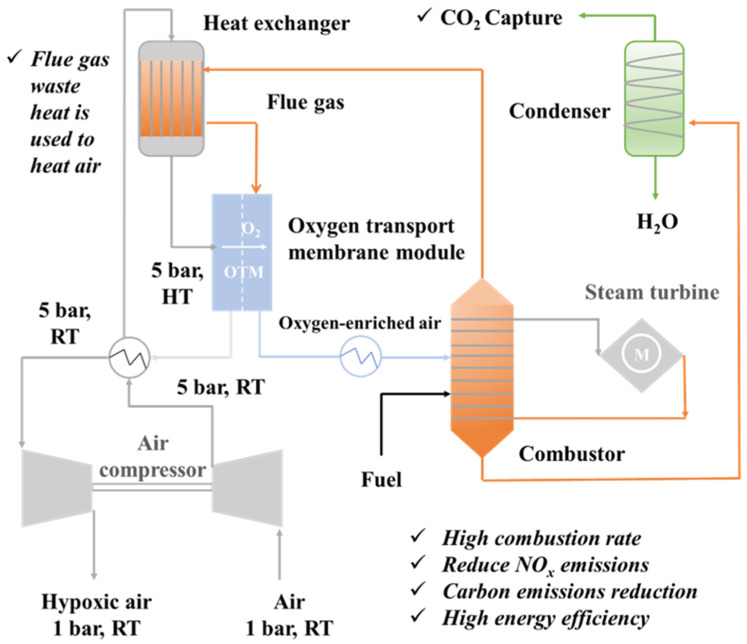
Schematic diagram illustrating the basic principle of CO_2_ capture via oxygen-enriched combustion using a mixed ion–electron conductor OTM.

Under oxygen-enriched combustion conditions, the harsh environment characterized by high temperatures, low oxygen partial pressure, flue gas corrosion, and long-term continuous operation places extremely high demands on oxygen-permeable membranes made of mixed conductors [110]. A rational membrane structure and fabrication process are the key to ensuring their successful engineering applications, as shown in Figure 8. The microscopic and macroscopic structures of the membrane directly determine its oxygen permeability and operational stability. A dense, defect-free matrix structure prevents gas leakage and ensures selective oxygen permeation. Controlling grain size, grain boundary structure, and pore distribution can optimize oxygen vacancy concentration and ion diffusion pathways, reduce oxygen transport resistance, and enhance oxygen permeability flux under oxygen-enriched conditions. Additionally, configurations such as gradient structures, support-dense composite membranes, and hollow fibers can balance mechanical strength with permeation area, mitigate issues related to thermal expansion mismatch and thermal stress cracking at high temperatures, and adapt to the long-term high-temperature operating environment of combustion systems.

## 6. Conclusions

Mixed ionic–electronic conducting (MIEC) oxygen transport membranes (OTMs) have become a core technology for efficient oxygen separation in the context of global carbon neutrality and energy transition, effectively compensating for the shortcomings of traditional oxygen production methods. This review systematically summarizes the oxygen transport mechanism, material system, structural design and preparation progress of MIEC OTMs. The oxygen permeation process is co-dominated by surface exchange reaction and bulk diffusion, and the rate-determining step is closely related to the characteristic thickness, which provides a theoretical basis for performance regulation. Single-phase materials represented by fluorite, perovskite and K_2_NiF_4_ types have achieved high oxygen flux through doping modification, but their stability in CO_2_-containing atmosphere is insufficient. Dual-phase composite membranes composed of ionic conductive phase and electronic conductive phase significantly improve chemical stability while maintaining permeability, becoming the mainstream development direction. In terms of structure, hollow fiber and asymmetric sandwich structure balance permeability and mechanical strength, and surface modification further optimizes transport kinetics. At present, the industrialization of OTMs is still restricted by the trade-off effect between permeability and stability, high operating temperature and scaling preparation.

The primary challenges currently hindering the large-scale commercialization of OTM include the difficulty of forming large, dense membranes; the susceptibility of sealing structures to failure under high-temperature and high-pressure conditions, resulting in high costs for maintaining airtightness; and the high cost of rare earth and precious metal-based raw materials, coupled with the high energy consumption of the sintering process, which makes the overall operating costs uncompetitive in the market. Future research should focus on multi-component co-doping, multi-scale structure design and integrated application with CCUS and hydrogen production systems, so as to promote the practical application of high-performance and high-stability MIEC oxygen permeable membranes.

## Figures and Tables

**Table 1:** The oxygen permeation flux models.

Models	Formulas	Classification	Ref.
Wagner	$J_{O_{2}}=\frac{1}{16F^{2}L}\int_{u_{O_{2}}^{\prime \prime }}^{u_{O_{2}}^{\prime }}t_{ion}t_{e}\sigma^{tot}du_{o_{2}}$	Bulk diffusion models	[29]
Bouwmeester	$J_{O_{2}}=\frac{1}{1+(2L_{C}/L)}\frac{1}{16F^{2}L}\int_{P_{o}^{\prime \prime }}^{P_{o}^{\prime }}\frac{\sigma_{e}\sigma_{ion}}{\sigma_{e}+\sigma_{ion}}d(\ln P_{O_{2}}^{*})$	Mixed factors limited	[30]
Xu-Thomson	$J_{O_{2}}=\frac{k_{r}/k_{f}(P_{O_{2}}^{\prime \prime -1/2}-P_{O_{2}}^{\prime -1/2})}{\left(1/k_{f}P_{O_{2}}^{\prime 1/2})+(2L/D_{v})+(1/k_{f}P_{O_{2}}^{\prime \prime 1/2}\right)}$	Surface reactions models	[31]
Li model	$J_{O_{2}}=\frac{A_{o}({P^{\prime \prime }}_{O_{2}}-{P^{\prime }}_{O_{2}})}{\left(1/k_{a})+(2L/D_{v})+(1/k_{d}\right)}$	Surface reactions models	[32]
Tan and Li	$J_{O_{2}}=\frac{k_{r}({P^{\prime }}_{O_{2}}^{1/2}-{P^{\prime \prime }}_{O_{2}}^{1/2})}{\frac{2k_{f}(R_{o}-R_{in}){P^{\prime }}_{O_{2}}^{1/2}{P^{\prime \prime }}_{O_{2}}^{1/2}}{D_{v}}+\frac{R_{m}{P^{\prime }}_{O_{2}}^{1/2}}{R_{in}}+\frac{R_{m}{P^{\prime \prime }}_{O_{2}}^{1/2}}{R_{o}}}$	Surface reactions models	[33]
Ghadimi	JO2=kr/kf([(a″+b″Re″c″)P″O2]−n−[(a′+b′Re′c′)P′O2]−n)(1/kf[(a′+b′Re′c′)P′O2]n)+(2L/Dv)+(1/kf[(a″+b″Re″c″)P″O2]n)	Surface reactions models	[34]
Kim model	$J_{O_{2}}=\frac{1}{4}\frac{\langle C_{ion}D_{a}\rangle }{L}\ln\frac{{P^{\prime }}_{O_{2}}}{{P^{\prime \prime }}_{O_{2}}}$	Chemical potential difference	[35]
Zhu model	$J_{O_{2}}=\frac{\Delta \mu_{O_{2}}^{tot}}{16F^{2}}\frac{1}{r^{tot}}$	Chemical potential difference	[36]
Van Hassel	$J_{O_{2}}=-kP_{O_{2}}^{n}(\frac{\Delta \mu }{RT})$	Effective medium approximation	[37]
D-G model	$J_{O_{2}}=\frac{k_{r1}((3-C_{v}^{\prime \prime }){\left[Fe_{Fe}^{*}\right]}_{sweep}^{2}-K_{1}P_{O_{2}}^{0.5}C_{v}^{\prime \prime }{\left[Fe_{Fe}^{X}\right]}_{sweep}^{2})}{2}$	Defect chemistry model	[38]

## Data Availability

No new data were created or analyzed in this study. Data sharing is not applicable to this article.
